# Why high-speed rail causes heterogeneous spatial patterns in firm innovation: Perspectives from intensive and extensive margins

**DOI:** 10.1371/journal.pone.0311621

**Published:** 2024-10-07

**Authors:** Longfei Zheng, Huasheng Zhu, Kwok Yuen Fan, Zheng Chang

**Affiliations:** 1 School of Geography, Faculty of Geographical Science, Beijing Normal University, Beijing, China; 2 Faculty of Business and Management, BNU-HKBU United International College, Zhuhai, China; The University of Tokyo, JAPAN

## Abstract

This study examines the impact of high-speed rail (HSR) on corporate innovation in China, analyzing county-level patent applications. Utilizing difference-in-differences regression, we break down the overall impact of HSR connectivity on innovation into two categories: extensive margins, where a greater number of firms become innovative, and intensive margins, where individual firms increase their level of innovation. HSR access has increased patent applications, particularly in manufacturing and non-high-tech services, affecting both margins. However, in high-tech services, the impact is significant only on the extensive margin. Effects vary between urban areas and peripheral counties, with knowledge spillovers and brain drain as key predictors.

## 1 Introduction

Innovation is widely recognized as a crucial driver for regional development [1]. Although the contribution of production factors like capital, labor, and land, as well as enhanced productivity, can stimulate economic growth, it is innovation that fuels sustainable development [2–4]. For emerging economies, innovation serves as a key mechanism for breaking free from the middle-income trap [5, 6]. As an example, China has implemented a variety of policy tools aimed at fostering innovation. Over the past decade, "mass entrepreneurship and innovation" has evolved into a pivotal national economic strategy [7]. The Global Innovation Index (GII), published by the World Intellectual Property Organization (WIPO), reveals that China’s ranking has climbed from 34th in 2012 to 11th in 2022.

A wealth of theoretical and empirical research underscores human capital and knowledge spillovers as pivotal catalysts for regional innovation and growth [8–10]. Transportation accessibility, a crucial determinant of human capital clustering and knowledge transfer, significantly influences innovation outcomes. Efficient transportation networks mitigate costs associated with human capital concentration and knowledge transfer, thereby fostering innovation [11]. Transportation systems serve dual functions: they bolster agglomeration benefits by making it easier for skilled workers to move into urban areas, especially metropolises [12], and they also cut the costs of knowledge and information flows among local innovators within regions [11]. Studies from China show that highways and railways notably contribute to innovation through facilitating knowledge spillovers and fostering skilled collaborations between cities [13, 14].

Among various transportation infrastructures, high-speed railways (HSRs) stand out for their role in enhancing mobility and enabling human interaction. Unlike conventional highways and railways primarily catering to cargo, HSRs are designed for passenger transport, operating at significantly higher speeds. China’s development of the world’s largest HSR network over the past two decades has made it a popular mode of intercity travel. Consequently, research has burgeoned, exploring its socioeconomic impacts, including economic growth, spatial expansion, housing and land markets, urban specialization, and sustainable development [15–20].

Recent scholarly focus has shifted towards examining HSR’s influence on urban innovation. Characterized by its emphasis on passenger transportation and high-speed operation, HSRs significantly affect intercity movement of skilled labor and dissemination of knowledge and information [13]. Empirical evidence suggests that HSR positively impacts regional innovation by facilitating knowledge spillovers [21, 22]. Previous research, including analyses of collaborative patents and academic publications, supports the notion that HSR enhances innovative outcomes by enabling face-to-face interactions and knowledge generation among skilled professionals, extending to innovation within manufacturing firms and the private sector [23, 24].

The expansion of HSR networks bears significant implications for spatial patterns of innovation. Theoretically, HSR construction can influence corporate innovation through two primary mechanisms. First, rapid transit facilitated by HSR enables highly-skilled workers to migrate more quickly, facilitating faster and broader dissemination of knowledge and ideas. At the intensive margin, this leads to increased innovation within individual firms. At the extensive margin, a richer knowledge environment encourages a greater number of firms to engage in innovation. Second, the core-peripheral theory from new economic geography predicts an urban agglomeration shadow, where growth in one area adversely affects neighboring regions [25, 26]. HSR can create such a shadow, impacting firm innovation in peripheral regions [27]. The network links metropolitan areas with peripheral counties, potentially causing a ’brain drain’ as highly-skilled workers seek opportunities in urban cores. Studies show this effect in both China and the United States, posing challenges for innovation [28, 29].

Knowledge spillover plays a crucial role in fostering innovation, both in urban core districts and peripheral regions. However, the brain drain effect, attributed to the agglomeration shadow, suggests that transportation accessibility may hinder innovation in peripheral areas. Resolving the conflicting predictions of these two channels requires robust empirical evidence. We utilize the construction of an HSR network as a quasi-experimental shock to explore the impact of transportation accessibility on innovation at the county level in China. This approach enables us to assess the divergent effects of transportation infrastructure on innovation between metropolitan and peripheral counties, shedding light on the relative merits of these theories.

Existing research has primarily focused on the manufacturing sector when studying the impact of HSR on firm innovation in China [23]. Yet, several critical questions remain unanswered. It is unclear whether innovation is predominantly driven by the extensive or intensive margin, and the relative influence of knowledge spillovers versus brain drain in peripheral regions remains ambiguous. Moreover, the effect of HSR on innovation in sectors beyond manufacturing remains underexplored. Addressing these gaps is essential for informing policymaking and supporting private enterprise.

Our study aims to fill these research gaps by employing data on patent applications and firm registration records in China from 2004 to 2016. Through a difference-in-differences (DID) empirical strategy, we investigate the heterogeneous effects of HSR on regional innovation outcomes. Additionally, we decompose these effects along both extensive and intensive margins, and across various regional and industrial dimensions. Furthermore, we examine the channels of heterogeneous HSR effects from the perspectives of firm location and knowledge spillover.

Our contributions to the literature are manifold. First, we comprehensively examine the causal effects of HSR on firm innovation at the county level in China, aligning with the core-periphery urban framework. Unlike previous studies conducted at the prefecture level, our research sheds new light on the heterogeneous effects of HSR connections within and between cities. Second, we decompose the total effects of HSR into extensive and intensive margins, identifying the primary drivers of HSR effects on innovation across different cities and industries, a novel approach in empirical research. Third, our study enriches the discourse on knowledge spillovers by exploring how geographical location and proximity influence knowledge dissemination. We find that the positive impact of HSR on innovation in counties of top-tier cities is primarily driven by the extensive margin, whereas in low-tier cities, it is driven by the intensive margin. Lastly, we contribute to the literature on proximity and innovation by uncovering the potentially detrimental effects of geographical closeness for firms located within the agglomeration shadow.

The remainder of this paper is organized as follows: Section 2 outlines the study’s background, focusing on HSR development and the patent system in China. Section 3 describes the research methodology and data sources. Section 4 presents the baseline empirical findings, while Section 5 discusses the observed heterogeneities. Finally, Section 6 offers conclusions and policy implications.

## 2 Background

### 2.1 HSR development

Over the past decade, China has constructed the most extensive HSR network in the world. The HSR system connects all cities with more than 500,000 inhabitants. The first HSR line was operated between Beijing and Tianjin with a speed of 350 km/h in 2008. By the end of 2020, the operating mileage of China’s HSR network was approximately 38,000 km, accounting for over the two-thirds of global HSR mileage [30].

HSR significantly increases proximity between cities by reducing travel costs in terms of both time and money. From the perspective of time cost, HSR networks have greatly increased travel speed and reduced time cost. According to the data from Ministry of Transport of the People’s Republic, at the beginning of the 21^st^ century, prior to HSR construction, the average speed of China’s conventional railway and highway systems were 65.7 km/h and 61 km/h respectively. In contrast, there was a 38,000 km HSR network in 2020 with an operating speed above 200 km/h. From a monetary perspective, the proportion of HSR fares among resident income is relatively low. In the 1990s, the fastest train from Beijing to Shanghai took 13.8 hours and the cost was more than 10% of the average monthly income of Beijing residents. However, in 2016, the HSR only required 4.5 hours for the same route and the cost was only 7% of the monthly income of Beijing residents. These data come from the 12306 China Railway (https://www.12306.cn/index/) and Beijing Municipal Bureau of Statistics (http://tjj.beijing.gov.cn/).

Based on the improved mobility provided by their high speeds, HSR networks provide significant opportunities for knowledge creation and innovation through interactions and learning among high-skilled workers. The flow of knowledge and information introduced by proximity makes workers more productive through face-to-face communications. Enhanced knowledge sharing and learning are reflected in the number of patent applications by firms.

### 2.2 Patent system in China

Patents are popular indicators of innovation outcomes [11, 31, 32]. China has established a mature patent system since the implementation of the Patent Law in 1985. In China, patents are divided into three categories: invention, utility model, and design. Among them, invention patents are more innovative and subject to rigorous examination for approval by the China National Intellectual Property Administration (CNIPA). The process of patent application and authorization in China mainly includes three steps: filing and sending the patent application documents, examination and approval by the CNIPA, and publication and authorization. The CNIPA lists newly authorized patents every week on its website (http://epub.cnipa.gov.cn/), which is the source of patent data used in this study.

**Fig 1:**
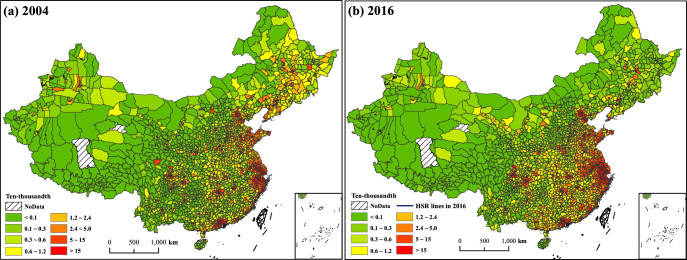
The distribution of patent applications in China, 2004 and 2016.

As a measure of innovation outcomes, the spatial distribution of patents is highly unbalanced and mainly concentrated in metropolitan cities. Fig 1 presents the spatial patterns of patent applications in 2004 and 2016, where there is a clear disparity in innovation outcomes across Chinese counties. The urban areas and counties with the highest innovation vitality are concentrated in the Yangtze River Delta, Greater Bay Area, and Beijing-Tianjin-Hebei urban agglomeration. These three urban agglomerations are the most economically prosperous regions in China, accounting for 37.4% of national economic outcomes in 2018 (Data are from National Bureau of Statistics of China). In 2004 (Fig 1A), when there was no HSR, the number of patent applications was concentrated in a few locations. Approximately 2.5% of urban areas and counties contributed over 50% of patent applications. By 2016 (Fig 1B), with the construction of an HSR network, innovation activities exhibited significant spatial spillovers. Approximately 50% of patent applications were produced in 4.1% of urban areas and counties.

Table 1 summarizes the types of applicants for Chinese invention patents over the past decade. Among all types of patent applications, firms play a dominant role and are the object of the empirical analysis conducted in this study. Furthermore, firm innovation behavior is more likely to be market driven compared to other types of patents. Therefore, understanding the patterns of patents applied for by firms is important for understanding the effects of HSR on innovation.

**Table 1:** Proportions of applicants for invention patents in China.

Year	Firm	University	Research institution	Individual
2020	60.9%	26.9%	7.1%	3.8%
2019	61.6%	25.3%	7.4%	4.6%
2018	64.3%	21.6%	5.9%	6.7%
2017	61.4%	23.1%	6.8%	7.2%
2016	62.7%	20.6%	6.7%	8.6%
2015	60.2%	21.7%	7.3%	9.3%
2014	56.5%	23.6%	8.3%	10.1%
2013	55.3%	23.2%	8.6%	11.6%
2012	54.7%	23.5%	7.8%	12.4%
2011	51.9%	23.7%	8.2%	15.4%

**Notes**: Data are from CNIPA.

## 3 Empirical strategy and data sources

Our empirical strategy utilizes three models to understand whether and how HSR expansion affects firm innovation. First, we estimate the causal effect of HSR connectivity on firm innovation outcomes at the county level by using a difference-in-differences (DID) model to measure the total effect of HSR. Next, we follow the literature and decompose the total effect of HSR on firm innovation into extensive and intensive margins [33, 34]. For the extensive margin, we estimate the impact of HSR connections on the number of firms with patent applications at the county level. For the intensive margin, we estimate the impact of HSR access on innovation intensity at the firm level.

### 3.1 Empirical strategy

First, we explore the aggregate effect of HSR connections on innovation outcomes from 2004 to 2016 at the county level. In mainland China, a prefecture-level city is typically composed of dozens of districts and counties. A county is typically located outside dense urban areas and can be considered as an internally integrated small market in terms of both work and life [35]. In contrast, a district is typically located within a dense urban area and a group of adjacent districts forms an integrated large market in a metro area. Therefore, we merge districts belonging to the same prefecture-level city into a single observation unit called an urban area. To be robust, we drop samples that were changed from county to urban district during the study period (2004 to 2016).

At the county level, HSR expansion can be considered as a quasi-experimental shock [36]. HSR is intended to reduce interregional travel time, particularly for large cities; however, HSR lines must pass through several intermediate cities. For those intermediate places, HSR shocks are largely exogenous [36, 37]. Following existing studies [37–39], we employ the standard two-way fixed effects DID model to quantify the total effects of HSR connections on the regional innovation outcomes at the county level. In recent years, there has been active research on the heterogeneous treatment effect (HTE) of the DID model (such as Chaisemartin and D’Haultfoeuille, 2020). De Chaisemartin and D’Haultfoeuille [40] showed that the Two-Way Fixed Effects (TWFE) DID method calculates the weighted average of individual treatment effects. They also highlighted a "negative weighting" issue, implying that a TWFE DID estimate could be negative even when all individual treatment effects are positive. These anomalies are more likely when treatments are introduced at varied intervals over an extended timeframe, leading to significant HTE. We evaluated the individual treatment effect weights and found all to be positive, which rules out the negative weighting problem.

In this model, counties with HSR access are defined as the treatment group, and all other counties are defined as the control group. The empirical model is defined in Eq (1):

$$Patent_{it}=\alpha_{0}+\alpha_{1}HSR_{it}+\alpha_{2}X_{it}+\mu_{i}+\delta_{pt}+\varepsilon_{it}$$ (1)

where ***Patent***_***it***_ is the natural logarithm of the number of approved patent applications in county *i* in year *t*. Owing to the existence of zero values, we add one to the number of patent applications and calculate the natural logarithm [13, 24, 41]. The term ***HSR***_***it***_ is a time-varying dummy variable that equals one if county *i* receives HSR service in year *t* and zero otherwise. In our multi-period DID model, ***HSR***_***it***_ is the interaction of the treatment and time dummy (treatment × after) in the basic DID model. ***X***_***it***_ is a vector of socioeconomic factors that includes both socioeconomic outcomes and natural environmental conditions, such as GDP, population, annual temperature, and average PM2.5 concentration of county *i* in year *t*. Both temperature and air quality are important factors in determining human interactions and knowledge exchanges. Barwick et al. show that HSR facilitates the use of intercity travel as an effective adaptation strategy to pollution and temperature extremes in China [42]. **μ**_**i**_ indicates county-level fixed effects, and **δ**_**pt**_ indicates province-by-year fixed effects. **ε**_**it**_ is a random error term. The coefficient α_1_ measures the total effects of HSR connections on the innovation outcomes. To address the spatial and serial correlation of the patent applications within a county, standard errors are clustered at the county level.

#### 3.1.1 Specification for the extensive margin

To explore the extensive margin of the HSR effect, the DID estimations are employed at the county level. In this specification, we are interested in whether more firms in a county become more innovative after the county is connected by HSR. The estimation for the extensive margin is specified as:

$$Firm_{it}=\alpha_{0}+\alpha_{1}HSR_{it}+\alpha_{2}X_{it}+\mu_{i}+\delta_{pt}+\varepsilon_{it}$$ (2)

where ***Firm***_***it***_ is the natural logarithm of the number of firms with at least one patent application in county *i* in year *t*. All other variables are exactly the same as those used in Eq (1). Standard errors are also clustered at the county level.

#### 3.1.2 Specification for the intensive margin

In this specification, we are interested in whether an existing firm located in county i becomes more innovative with more patent applications after the county is connected by HSR. To explore the intensive margin of the HSR effect, we conduct the DID estimations at the firm level, as shown in Eq (3).

$$Patent_{ijt}=\alpha_{0}+\alpha_{1}HSR_{it}+\tau_{j}+\delta_{pt}+\varepsilon_{it}$$ (3)

where ***Patent***_***ijt***_ is the number of patent applications by firm *j* in county *i* in year *t*. We control for firm-level fixed effects, **τ**_**j**_, which absorbs time-invariant firm characteristics. The terms ***HSR***_***it***_, **δ**_**pt**_, and **ε**_**it**_ are exactly the same as those used in Eq (1). Standard errors are clustered at the firm level.

### 3.2 Data and summary statistics

The data used in this study are obtained from multiple sources. The first dataset is composed of patent applications and publication data from the CNIPA (http://epub.cnipa.gov.cn/). This dataset includes all patent information, including category, application ID and date, publication ID and date, applicant, and location. From 2004 to 2016, there were a total of 13.5 million patent application records. The proportion of invention patents is 34.56%, which is the main measure of innovation considered in this study. Based on the categories and locations of patents, we aggregate the firm patent records at the county level.

The second dataset contains firm registration data from the State Administration for Market Regulation of China (http://wsdj.samr.gov.cn/saicmccx/), which includes firm registration information, such as name, industry, date of registration, and location. We decompose the total effect of HSR into extensive and intensive margins by linking the patent and firm databases based on the names of applicants and firms. This study focuses on patents filed by firms, which account for more than 60% of invention patents. At the county level, we count the annual total number of firms with patent application records to measure the extensive margin. At the firm level, we calculate the annual total number of patent applications for each firm to measure the intensive margin.

The third dataset consists of geocoded HSR stations and lines, which are obtained from multiple sources. We obtain the HSR network data covering the period of 2008 to 2016 from railway train-schedule yearbooks, Harvard Dataverse [43], and 12306 China Railway (www.12306.cn/index/index.html) and conduct cross-validation. We are able to match the location of each HSR stations to each county and obtain the opening times of HSR stations from the Railway Statistical Yearbook of China.

Finally, socioeconomic data at the county level are collected from the China County Statistical Yearbook, and the time-varying indicators of natural conditions are obtained from the Institute of Geographic Sciences and Natural Resources Research (https://www.resdc.cn/). The annual PM2.5 concentrations are obtained from the NASA Socioeconomic Data and Applications Center (SEDAC), and we calculate the annual average of PM2.5 concentrations at the county level using the ArcGIS software.

Table 2 presents variable definitions and summary statistics, and is organized into three panels. Panel A presents the three dependent variables, which are used to estimate the total effect, extensive margin, and intensive margin of HSR on patent applications in our regressions, respectively. All numbers are relatively high for counties with HSR connections compared to counties without HSR connections. Panel B summarizes the statistics for the independent variables at the county and firm levels, which are time-varying treatment dummies. Panel C presents statistics on control variables, including GDP, population, and annual average of PM2.5 concentration and temperature at the county level. Again, these economic and demographic outcomes are higher in regions with HSR connections compared to other regions.

**Table 2:** Variable definition and summarized statistics.

Variable	Scale	Description	Mean and standard deviation		
			Full Sample	With HSR	Without HSR
Panel A: Dependent variables					
Patent number (Total effect)	County level	Total number of invention patent applications by firms in each county-year	67.83	284.63	13.08
			(747.7)	(1632.51)	(111.67)
W_Citation (Total effect)	County level	Total number of invention patent applications weighted by citation	86.2 (889.03)	526.03 (2329.04)	15.93 (119.88)
Firm number (Extensive margin)	County level	Total number of firms with invention patent application in each county-year	13.62	55.3	3.09
			(129.33)	(282.38)	(16.37)
Patent intensity (Intensive margin)	Firm level	Number of invention patent applications in each firm-year	1.05	1.09	0.88
			(8.94)	(9.69)	(4.36)
Panel B: HSR status (Independent variables)					
HSR	County level	Dummy, 1 for county after operation of HSR, 0 otherwise	0.062	0.305	0
			(0.24)	(0.461)	(0)
HSR	Firm level	Dummy, 1 for firm located in the county with HSR access, 0 otherwise	0.51	0.627	0
			(0.5)	(0.484)	(0)
Panel C: Socio-economic outcomes and natural conditions (Control variables)					
GDP	County level	Gross Domestic Product (billion yuan)	21.81	64.59	11.01
			(84.99)	(179.85)	(17.4)
Pop	County level	Number of permanent residents (thousand)	615	1096.8	493.34
			(799.86)	(1527.47)	(372.6)
PM2.5	County level	Annual surface of PM2.5 concentration (μg/m3)	34.7	40.18	33.36
			(18.23)	(15.35)	(18.62)
Temperature	County level	Annual average temperature of the county (°C)	13.73	15.76	13.23
			(5.51)	(4.72)	(5.57)
Obs	County level	Number of observations	25,857	5,213	20,644
Obs	Firm level	Number of observations	1,537,488	1,249,485	288,003

Notes: Standard deviations are in parenthesis. The total effect and extensive margin of HSR impacts are measured at the county level, and the intensive margin of HSR impacts is measured at the firm level.

### 3.3 Stylized facts

**Fig 2:**
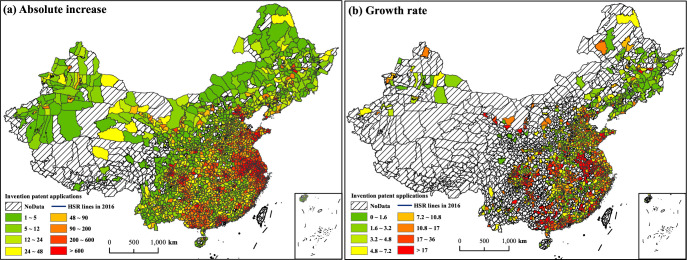
The spatial distribution of patent application growth from 2007 to 2016. Notes: In Fig 2(B), the growth rate = (Invention patent application in 2016 / Invention patent application in 2007)-1. In order to avoid the extremely large growth due to the small value of denominator, we set a threshold of 10 for the 2007 patent application. Counties are not included in Fig 2(B) if their 2007 invention patent application is less than 10.

Before diving into the empirical results, we combined the county-level patent and HSR expansion data to exhibit the stylized facts. China’s HSR network construction began in 2008. We calculate the absolute increase and growth rate of the number of invention patent applications in counties from 2007 to 2016, as shown in Fig 2. Based on the absolute increment in Fig 2(A), we find that the growth in invention patents is concentrated in metropolises and their geographical vicinity. Fig 2(B) presents the growth rate, which is defined as net growth divided by the number of patent applications in 2007. The fastest-growing regions are concentrated around HSR network hubs. This pattern suggests that spatial patterns of innovation activity are likely to respond positively to HSR access. This evidence is suggestive, and we will explore the causal effects of HSR access on regional innovation in the next section.

## 4 Baseline results

### 4.1 Baseline estimations of total effect

We employ Eq (1) to estimate the causal effects of HSR connections on invention patent applications, and the baseline results are shown in Table 3. The dependent variable is the natural logarithm of the total number of patent applications for each county-year. Column (1) presents the results of the pooled ordinary least squares (OLS) regression and the coefficient of HSR is significantly positive, suggesting that HSR connections are positively correlated with regional innovation at the county level. Columns (2) and (3) correspond to DID regressions with two-way fixed effects. The results show that the total effects of HSR access on regional innovation outcomes are significantly positive, with a marginal effect of 43.1%. Given concerns regarding the robustness of our data and model, we weight the number of patents with citations and re-run our regressions. The results based on the weighted patent numbers are shown in the last three columns, which are largely consistent with the results in the first three columns.

**Table 3:** Baseline DID regression results of total effects.

	Patents			Patents weighted by citation (W_Citation)		
	OLS	DID		OLS	DID	
	(1)	(2)	(3)	(4)	(5)	(6)
HSR	1.203***	0.438***	0.431***	1.153***	0.375***	0.371***
	(0.0850)	(0.0439)	(0.0445)	(0.0875)	(0.0442)	(0.0450)
ln(GDP)	0.980***		-0.00827	1.049***		-0.00396
	(0.0254)		(0.0482)	(0.0263)		(0.0519)
ln(Pop)	-0.301***		0.362***	-0.321***		0.305***
	(0.0400)		(0.0925)	(0.0418)		(0.0940)
ln(Temperature)	0.460***		0.0929	0.503***		0.0428
	(0.0805)		(0.294)	(0.0851)		(0.318)
ln(PM2.5)	-0.118***		-0.224***	-0.107**		-0.24***
	(0.0416)		(0.0641)	(0.0439)		(0.07)
County fixed effects	No	Yes	Yes	No	Yes	Yes
Province-Year fixed effects	No	Yes	Yes	No	Yes	Yes
Number of counties/firms	-	1,986	1,972	-	1,986	1,972
R^2^	0.569	0.869	0.869	0.562	0.852	0.853
Observation	25,675	25,818	25,636	25,675	25,818	25,636

Notes: *, **, *** refers to the statistical significance at 10%, 5%, and 1%, respectively. The standard errors are reported in parentheses and clustered at county level.

### 4.2 Extensive versus intensive margin of the HSR effect

Table 3 presents the total effect of HSR on regional innovation growth, where the apparent surge may be driven by two different margins. To examine the extensive and intensive margins of the HSR effect, we run the DID models in Eqs (2) and (3) at the county and firm levels, respectively. Table 4 presents the results.

**Table 4:** Baseline DID regression results of extensive and intensive margin.

	Total effect		Extensive margin	Intensive margin	
	Patent number	W_Citation	Firm number with patent	Innovation intensity	W_Citation
	(1)	(2)	(3)	(4)	(5)
HSR	0.431***	0.371***	0.331***	0.144***	0.150***
	(0.0445)	(0.0450)	(0.0288)	(0.0286)	(0.0367)
Controls	Yes	Yes	Yes	-	-
County/firm fixed effects	Yes	Yes	Yes	Yes	Yes
Province-Year fixed effects	Yes	Yes	Yes	Yes	Yes
Number of counties/firms	1,972	1,972	1,972	174,542	174,542
R^2^	0.869	0.853	0.892	0.424	0.447
Observation	25,636	25,636	25,636	1,531,946	1,531,946

Notes: *, **, *** refers to the statistical significance at 10%, 5%, and 1%, respectively. The standard errors are reported in parentheses. For total effect and extensive margin, the standard errors are clustered at county level. For the intensive margin, the standard errors are clustered at firm level.

Columns (1) and (2) present the previous total effect of HSR as a reference. Column (3) presents the results for the extensive margin, and one can see that HSR increases the number of firms with patent applications by an average of 33.1%. At the intensive margin, the number of patent applications by each firm increases by 0.144, as shown in column (4). As a robustness check, we run regressions weighted by citations for innovation intensity at the firm level. The results are listed in column (5) and are largely consistent with the estimations in column (4). In summary, the positive total effect of HSR on innovation outcomes at the county level is driven by both the extensive and intensive margins.

### 4.3 Robustness checks

The baseline results demonstrate that HSR connections have a significant positive impact on firms’ innovation. In this section, we present additional robustness checks to verify the reliability of the baseline results.

**Fig 3:**
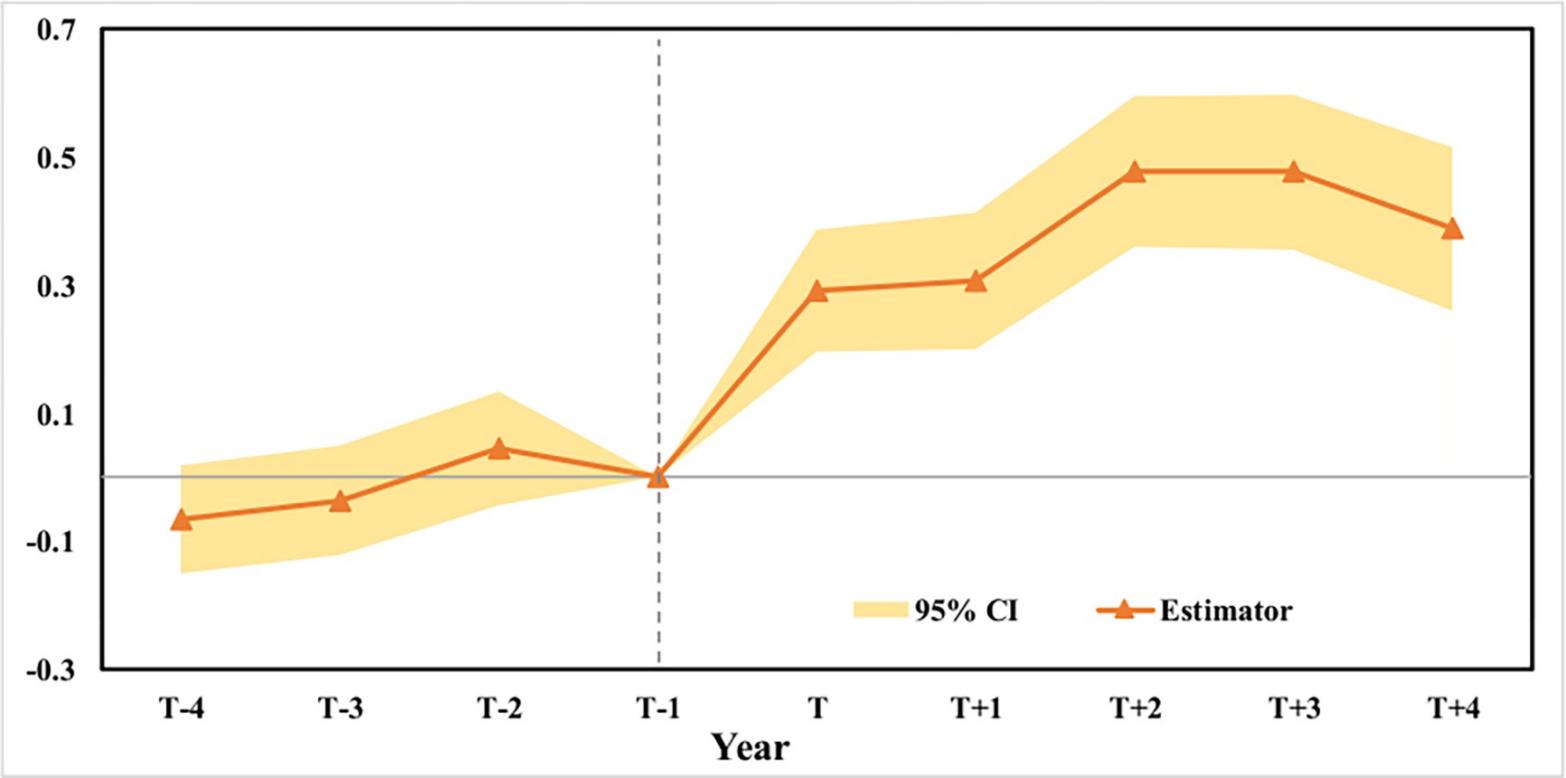
Results of event study. Notes: The horizontal axe shows the event-study dummies for each year, and the vertical axe indicates the effect of HSR on innovation outcomes in each year relative to the reference year (T-1).

**Fig 4:**
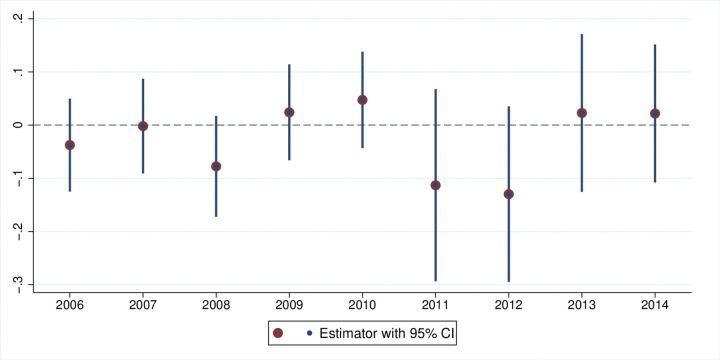
Placebo test.

1. Validation of the parallel trends assumption. One prerequisite for a valid DID estimation is that the parallel trends assumption must hold during the pre-treatment period. In other words, the growth of innovation outcomes in the treatment and control counties should maintain similar trends prior to HSR connection. To verify the parallel trends assumption, we follow previous studies [12, 38, 39] and conduct a standard event study to determine the coefficients within a 9-year event window. In the event study framework, a set of pre- and lag-year dummies of actual HSR connections is generated to test the significance of the regression coefficients of these dummies. The model is specified as follows:
$$Y_{it}=\beta_{0}+\sum_{j=-4}^{4}\beta_{i}HSR_{j}\times 1[j=T]+\alpha_{2}X_{it}+\mu_{i}+\delta_{pt}+\varepsilon_{it}$$ (4)
  where 1[j = T] is an event study year dummy that equals one for each pre- and lag-year before and after HSR connection. All other variables are exactly the same as those used in Eq (1) for the DID model. We consider the year before HSR connection (*j* = *T-1*) as a reference, and the coefficients for other years with corresponding 95% confidence intervals are plotted in Fig 3. The results presented in Fig 3 support the validity of the DID estimations when assuming parallel trends. The coefficients before year T-1 are not significantly different from zero, which demonstrates that the growth trends of innovation outcomes in the treatment and control counties do not exhibit significant differences prior to HSR connection.
2. Placebo Test: We follow the approach outlined by Zheng et al. [20] to randomly select a year between 2006 and 2014 as the hypothetical start year for HSR operation. We then rerun the DID model to estimate HSR’s total impact on firm innovation, based on this hypothetical start year. The results are summarized in Fig 4. This figure also displays the 5% significance interval, represented by the upper and lower bounds of the coefficients. None of the results cross the 5% significance threshold, further corroborating the robustness of our baseline estimation.
3. Dropping the samples of large cities. Although the parallel trends assumption is supported by the event study, it is still possible that the public sector prefers to connect to metropolises through HSR lines, where there is a large demand for face-to-face communication. Selection bias in HSR setting can lead to spurious or overestimated HSR impacts [12]. To rule out this possibility, we drop the sample of metropolises for our regressions. Following previous studies [12], we run regressions by excluding first- and second-tier cities in China, and the results are presented in Table 5. The structure in Table 5 is identical to that in Table 4. The results are largely consistent with the baseline results, confirming that our estimations are not affected by selection bias.

**Table 5:** DID Estimations by excluding large cities.

	Total patent		Extensive margin	Intensive margin	
	Patent number	W_Citation	Firm number with patent	Innovation intensity	W_Citation
	(1)	(2)	(3)	(4)	(5)
HSR	0.408***	0.350***	0.309***	0.114***	0.134***
	(0.0456)	(0.0460)	(0.0298)	(0.0386)	(0.0450)
Controls	Yes	Yes	Yes	-	-
County/firm fixed effects	Yes	Yes	Yes	Yes	Yes
Province-Year fixed effects	Yes	Yes	Yes	Yes	Yes
Number of counties/firms	1,900	1,900	1,900	72,266	72,266
R^2^	0.854	0.836	0.873	0.316	0.337
Observation	24,700	24,700	24,700	655,419	655,419

Notes: *, **, *** refers to the statistical significance at 10%, 5%, and 1%, respectively. The standard errors are reported in parentheses and clustered at county and firm levels.

## 5 Spatial and industrial heterogeneities

The baseline results in the previous section represent the average effect of HSR on innovation, but patterns could differ between urban areas and peripheral counties in different industrial sectors.

### 5.1 Urban areas versus peripheral counties

Previous studies have found that HSR promotes the growth of large cities at the expense of nearby small cities. After the HSR connection, more people moved from peripheral counties to urban areas, which may affect the spatial pattern of patent applications [12, 38]. To understand the heterogeneous effects of HSR on innovation in urban areas and peripheral counties, we consider the interaction of the HSR dummy with urban and county indicators and execute DID regressions by employing Eqs (1) to (3). The results are presented in Table 6.

**Table 6:** The disparity between urban areas and peripheral counties.

	Total effect		Extensive margin	Intensive margin	
	Patent number	W_Citation	Firm number with patent	Innovation	W_Citation
	(1)	(2)	(3)	(4)	(5)
HSR×Urban	0.686***	0.556***	0.565***	0.176***	0.174***
	(0.0602)	(0.0622)	(0.0368)	(0.0321)	(0.0412)
HSR×County	0.269***	0.254***	0.183***	-0.0243	0.0268
	(0.0550)	(0.0563)	(0.0348)	(0.0372)	(0.0437)
Controls	Yes	Yes	Yes	-	-
County/firm fixed effects	Yes	Yes	Yes	Yes	Yes
Province-Year fixed effects	Yes	Yes	Yes	Yes	Yes
Number of counties/firms	1,972	1,972	1,972	174,542	174,542
R^2^	0.87	0.853	0.893	0.424	0.447
Observation	25,636	25,636	25,636	1,531,946	1,531,946

Notes: *, **, *** refers to the statistical significance at 10%, 5%, and 1%, respectively. The standard errors are reported in parentheses and clustered at county and firm levels.

The organization in Table 6 is similar to that in Table 4 for the baseline results. Column (1) reveals that HSR access has a significantly positive effect on innovation outcomes in both urban areas and v counties, with coefficients of 68.6% and 26.9%, respectively. The second column uses the patent citations to weight the dependent variable, and the results remain largely consistent. The regression coefficients in the first two columns are statistically significant at the 1% level. Column (3) presents the estimation of the extensive margin. One can see that the number of firms with patent applications in both urban areas and peripheral counties connected to HSR networks increases significantly, but the effects in urban areas are much greater than those in peripheral counties. Columns (4) and (5) present estimates for the intensive margin. The results indicate that firms in urban areas experience a significant increase in innovation intensity after connecting to the HSR network, whereas firms in peripheral counties experience no significant change with HSR access. In summary, the positive effects of HSR on innovation in urban areas is driven by both the extensive and intensive margins, whereas the positive effect of HSR in peripheral counties is driven only by extensive margin.

### 5.2 Heterogeneities by economic sectors

Next, we examine the impact of HSR connections on innovation outcomes in different economic sectors. The firms in our dataset can be largely classified into the manufacturing and service sectors. The service sector can be further categorized into high-tech and other service industries. The high-tech service industry consists of the IT industry, and scientific research and technology service industry. Considering the knowledge intensity across sectors and passenger transport characteristics of HSR, we expect that the service industry, particularly the high-tech service industry, will benefit the most from HSR access. To explore the heterogeneous effects across sectors, we execute DID regressions at the county and firm levels. The results are presented in Table 7.

**Table 7:** Regression results by sectors.

	Patent number (Total)				Firm number with patent (Extensive margin)				Innovation intensity (Intensive margin)			
	Manufacturing	Service	High-tech service	Other service	Manufacturing	Service	High-tech service	Other service	Manufacturing	Service	High-tech service	Other service
	(1)	(2)	(3)	(4)	(5)	(6)	(7)	(8)	(9)	(10)	(11)	(12)
HSR	0.460***	0.621***	0.621***	0.499***	0.324***	0.428***	0.434***	0.326***	0.203***	0.117**	0.0292	0.198***
	(0.0467)	(0.0520)	(0.0517)	(0.0488)	(0.0298)	(0.0342)	(0.0349)	(0.0304)	(0.0387)	(0.0468)	(0.0736)	(0.0623)
Controls	Yes	Yes	Yes	Yes	Yes	Yes	Yes	Yes	-	-	-	-
County/firm fixed effects	Yes	Yes	Yes	Yes	Yes	Yes	Yes	Yes	Yes	Yes	Yes	Yes
Province-Year fixed effects	Yes	Yes	Yes	Yes	Yes	Yes	Yes	Yes	Yes	Yes	Yes	Yes
Number of counties/firms	1,972	1,972	1,972	1,972	1,972	1,972	1,972	1,972	79,837	86,074	59,739	26,335
R^2^	0.843	0.786	0.772	0.747	0.867	0.827	0.818	0.781	0.465	0.408	0.414	0.369
Observation	25,636	25,636	25,636	25,636	25,636	25,636	25,636	25,636	803,561	651,232	428,833	222,399

Notes: *, **, *** refers to the statistical significance at 10%, 5%, and 1%, respectively. The standard errors are reported in parentheses and clustered at county and firm levels. The high-tech service industry consists of the information technology industry and scientific research and technology service industry.

In Table 7, one can see that the HSR connectivity has a significant positive effect on innovation outcomes in all sectors, as shown in columns (1) to (4). Elasticity is relatively large for the high-tech service sector and smaller for the manufacturing sector. Columns (5) to (8) correspond to the extensive margin and exhibit patterns similar to the total effect. However, we find different patterns for the intensive margin in columns (9) to (12). At the intensive margin, HSR has a significant positive impact on the innovation intensity of the manufacturing and non-high-tech service industries. Furthermore, elasticity is large for manufacturing and smaller for services. On average, the number of invention patent applications of manufacturing firms increases by 0.203 following the connection of the HSR network, whereas that of the service firms only increases by 0.12.

We further explore the heterogeneous effects of HSR between urban areas and counties areas across the sectors, as shown in Table 8. Similar to the findings in Table 7, columns (1) to (4) in Table 8 indicate that HSR access has a significant positive effect on innovation outcomes across all sectors, both in urban areas and peripheral counties. However, the magnitudes in the urban areas are much greater than those in peripheral counties in all sectors. Elasticity is relatively large for the high-tech service sector and smaller for the manufacturing sector. Columns (5) to (8) present the estimates for the extensive margin of the HSR effect, which exhibits patterns similar to the total effect. However, we find different patterns for the intensive margin in columns (9) to (12). In urban areas, HSR has a significant positive impact on the innovation intensity of firms in the manufacturing and non-high-tech service industries, which is consistent with the findings in Table 7. In peripheral counties, HSR-induced intensive margin growth appears only for firms in the non-high-tech service sector. Additionally, HSR has actually led to a significant reduction in the innovation intensity of high-tech service firms.

**Table 8:** The urban and county disparity by sectors.

	Patent number (Total)				Firm number with patent (Extensive margin)				Innovation intensity (Intensive margin)			
	Manufacturing	Service	High-tech service	Other service	Manufacturing	Service	High-tech service	Other service	Manufacturing	Service	High-tech service	Other service
	(1)	(2)	(3)	(4)	(5)	(6)	(7)	(8)	(9)	(10)	(11)	(12)
HSR×Urban	0.871***	1.285***	1.302***	1.090***	0.625***	0.937***	0.948***	0.760***	0.264***	0.130***	0.0619	0.188***
	(0.0656)	(0.0772)	(0.0812)	(0.0787)	(0.0385)	(0.0528)	(0.0569)	(0.0493)	(0.0455)	(0.0496)	(0.0795)	(0.0649)
HSR×County	0.199***	0.198***	0.188***	0.124***	0.133***	0.105***	0.107***	0.0503**	-0.0449	0.00299	-0.215**	0.307**
	(0.0524)	(0.0504)	(0.0468)	(0.0454)	(0.0339)	(0.0281)	(0.0273)	(0.0242)	(0.0437)	(0.0763)	(0.0878)	(0.133)
Controls	Yes	Yes	Yes	Yes	Yes	Yes	Yes	Yes	-	-	-	-
County/firm fixed effects	Yes	Yes	Yes	Yes	Yes	Yes	Yes	Yes	Yes	Yes	Yes	Yes
Province-Year fixed effects	Yes	Yes	Yes	Yes	Yes	Yes	Yes	Yes	Yes	Yes	Yes	Yes
Number of counties/firms	1,972	1,972	1,972	1,972	1,972	1,972	1,972	1,972	79,837	86,074	59,739	26,335
R^2^	0.844	0.795	0.783	0.757	0.869	0.839	0.831	0.795	0.465	0.408	0.414	0.369
Observation	25,636	25,636	25,636	25,636	25,636	25,636	25,636	25,636	803,561	651,232	428,833	222,399

Notes: *, **, *** refers to the statistical significance at 10%, 5%, and 1%, respectively. The standard errors are reported in parentheses and clustered at county and firm levels.

### 5.3 Heterogeneities by urban hierarchies

The HSR network connects cities at different levels of development and the impact of HSR on innovation may vary with the level of urban development. In China, cities are ranked hierarchically according to their level of development [30]. The first- and second-tier cities are mainly the centrally administrated municipalities and provincial capital cities with prosperous economies, such as Shanghai, Guangzhou, and Zhengzhou, which are defined as “top-tier” cities. The other cities are mainly small- and medium-sized cities in development and are defined as “low-tier” cities in this study. To explore the different patterns between city tiers, we run DID regressions by interacting the HSR dummy with the city tier and urban/county indicator. Table 9 summarizes the estimation results.

**Table 9:** Heterogeneities by urban hierarchies.

	Patent number (Total)				Firm number with patent (Extensive margin)				Innovation intensity (Intensive margin)			
	Manufacturing	Service	High-tech Service	Other service	Manufacturing	Service	High-tech service	Other service	Manufacturing	Service	High-tech service	Other service
	(1)	(2)	(3)	(4)	(5)	(6)	(7)	(8)	(9)	(10)	(11)	(12)
HSR x Top tier x Urban	0.906***	1.874***	1.955***	1.694***	0.723***	1.498***	1.570***	1.269***	0.256***	0.100*	0.0155	0.160**
	(0.116)	(0.102)	(0.101)	(0.104)	(0.0669)	(0.0603)	(0.0608)	(0.0614)	(0.0509)	(0.0531)	(0.0862)	(0.0683)
HSR x Top tier x County	0.398***	0.556***	0.531***	0.278**	0.329***	0.336***	0.344***	0.149**	-0.206***	-0.201**	-0.35***	0.0799
	(0.0971)	(0.125)	(0.119)	(0.109)	(0.0676)	(0.0719)	(0.0702)	(0.0646)	(0.0540)	(0.0907)	(0.0963)	(0.191)
HSR x Low tier x Urban	0.856***	0.987***	0.972***	0.781***	0.578***	0.652***	0.632***	0.499***	0.279***	0.277**	0.297**	0.304*
	(0.0752)	(0.0835)	(0.0891)	(0.0896)	(0.0432)	(0.0492)	(0.0531)	(0.0496)	(0.104)	(0.110)	(0.146)	(0.161)
HSR x Low tier x County	0.132**	0.0802	0.0742	0.0739	0.0670*	0.0285	0.0287	0.0191	0.202***	0.307**	0.0982	0.500***
	(0.0607)	(0.0496)	(0.0452)	(0.0477)	(0.0376)	(0.0263)	(0.0254)	(0.0229)	(0.0669)	(0.125)	(0.175)	(0.174)
Controls	Yes	Yes	Yes	Yes	Yes	Yes	Yes	Yes	-	-	-	-
County/firm fixed effects	Yes	Yes	Yes	Yes	Yes	Yes	Yes	Yes	Yes	Yes	Yes	Yes
Province-Year fixed effects	Yes	Yes	Yes	Yes	Yes	Yes	Yes	Yes	Yes	Yes	Yes	Yes
Number of counties/firms	1972	1972	1972	1972	1972	1972	1972	1972	1972	86074	59739	26335
R^2^	0.844	0.798	0.787	0.761	0.869	0.845	0.838	0.801	0.465	0.408	0.414	0.369
Observation	25636	25636	25636	25636	25636	25636	25636	25636	25636	651232	428833	222399

Notes: *, **, *** refers to the statistical significance at 10%, 5%, and 1%, respectively. The standard errors are reported in parentheses and clustered at county and firm levels.

In Table 9, four interactions are used to explore the heterogeneity of the HSR effect in four types of counties/districts: urban areas of top-tier cities (HSR x Top tier x Urban), urban areas of low-tier cities (HSR x Low tier x Urban), peripheral counties of top-tier cities (HSR x Top tier x County), and peripheral counties of low-tier cities (HSR x Low tier x County). We find that innovation outcomes increase across all sectors following HSR connection in urban areas in both top- and low-tier cities. Both the extensive and intensive margins contribute significantly to the total effect.

In contrast, the patterns of HSR effects in peripheral counties are mixed. In peripheral counties of top-tier cities, HSR access has a positive impact on innovation outcomes in all industries and the extensive margin of growth contributes to the positive total effect. Regarding the intensive margin, HSR connectivity has a significant negative impact on the innovation intensity of manufacturing and high-tech service firms. In peripheral counties of low-tier cities, HSR access only has a positive impact on the innovation of manufacturing firms. We decompose the total effects into extensive and intensive margins and find that the intensive margin plays a dominant role in patent applications for firms in peripheral counties of low-tier cities.

### 5.4 Understanding the heterogeneities

How can the sectoral and spatial heterogeneity of the HSR effect be explained? As discussed in Section 1, the impact of HSR on regional innovation depends on the trade-off between the effects of knowledge spillover and brain drain. The empirical results indicate that HSR has a positive impact on innovation in most regions and industries. Table 9 reveals that all coefficients are positive in urban districts. Therefore, we can conclude that knowledge spillover, rather than brain drain, plays a dominant role in explaining the positive impact of HSR on the number of patent applications in urban areas.

For peripheral counties, we observe a mixture of knowledge spillover and brain drain effects. Peripheral counties in top-tier cities are geographically adjacent to metropolises, where both knowledge spillover and brain drain effect can play a role in firm innovation. On the one hand, by connecting urban areas to peripheral counties using HSR, firms may rebalance the trade-off between the high revenue and high cost of urban areas, and low revenue and low cost of peripheral counties [12]. The low land and labor costs of peripheral counties provide an excellent environment for knowledge-based entrepreneurial activities. This explains the significant positive coefficients of the extensive margin in Table 9. On the other hand, the core-periphery theory suggests the likelihood of a ‘brain drain’ effect. Specifically, high skilled workers are more likely to migrate from small counties to metropolises in search of opportunities and wage premiums [30]. This accelerates the brain drain in peripheral counties. For each firm, innovation intensity declines with the loss of highly skilled workers, which explains the negative coefficients of the intensive margin estimates.

In contrast, in low-tier cities, the urban districts are more attractive for firm establishment (extensive margin) compared to counties. However, each firm tends to innovate more in both urban districts and counties due to knowledge spillover. There is no brain drain effect because the urban core in low-tier cities is not sufficiently strong to attract talent away from peripheral counties.

## 6 Conclusion

This study explores the causal effects of HSR connectivity on regional innovation outcomes in China and decomposes the total effects into extensive and intensive margins through a set of DID regressions. We demonstrate that HSR connectivity leads to a patenting surge at the county level. For manufacturing and non-high-tech service industries, the rising firm innovation is jointly driven by the extensive and intensive margins. For high-tech service firms, innovation is driven largely by the extensive margin. Spatially, the extensive and intensive margins jointly contribute to the positive effect of HSR on firms in urban areas, whereas the impacts of HSR in peripheral counties are mixed.

To understand these patterns, we consider the knowledge spillover and brain drain effects to predict the total effects of HSR from two opposing channels. We find that in most regions, knowledge spillover can better predict the significant positive effects of HSR. However, knowledge spillover and brain drain jointly predict the innovation outcomes in peripheral counties of top-tier cities. Knowledge spillover explains the positive effect at the extensive margin because firms can easily learn from each other given the geographical proximity. However, the brain drain effect explains the negative effect at the intensive margin as peripheral firms lose highly skilled workers, which can reduce the innovation intensity.

The implications of this study are multifold and hold substantial weight for policymakers, especially those in transportation and economic development sectors: First, this study empirically proves that HSRs have a positive impact on regional innovation. This validates the need for continued investment in HSR infrastructure but with a nuanced understanding of its differential impacts at the county level and across various economic sectors. Second, this study underscores the heterogeneity of HSR’s effects, revealing that positive impacts on innovation are more pronounced in top-tier cities through the extensive margin and in low-tier cities through the intensive margin. This insight can guide policymakers in tailoring region-specific strategies for enhancing innovation. Third, while HSR connectivity offers several advantages, it also poses the risk of ’brain drain’ from peripheral regions to metropolitan areas. Policymakers need to be aware of this effect and perhaps develop compensatory measures, such as incentives for firms or skilled workers to remain in or relocate to peripheral areas. Fourth, given that HSR aligns well with the core-periphery urban framework, policymakers should consider this dimension in HSR route planning and regional economic development strategies. Policies could aim to minimize the agglomeration shadow effects that could stifle innovation in adjacent regions.
